# Sequence Variations in the Flagellar Antigen Genes *fliC*_H25_ and *fliC*_H28_ of *Escherichia coli* and Their Use in Identification and Characterization of Enterohemorrhagic *E. coli* (EHEC) O145:H25 and O145:H28

**DOI:** 10.1371/journal.pone.0126749

**Published:** 2015-05-22

**Authors:** Lothar Beutin, Sabine Delannoy, Patrick Fach

**Affiliations:** 1 National Reference Laboratory for *Escherichia coli*, Federal Institute for Risk Assessment (BfR), Diedersdorfer Weg 1, D-12277, Berlin, Germany; 2 Université Paris-Est, Anses (French Agency for Food, Environmental and Occupational Health and Safety), Food Safety Laboratory, IdentyPath platform, 14 rue Pierre et Marie Curie, Fr-94700, Maisons-Alfort, France; USDA-ARS-ERRC, UNITED STATES

## Abstract

Enterohemorrhagic *E*. *coli* (EHEC) serogroup O145 is regarded as one of the major EHEC serogroups involved in severe infections in humans. EHEC O145 encompasses motile and non-motile strains of serotypes O145:H25 and O145:H28. Sequencing the *fliC*-genes associated with the flagellar antigens H25 and H28 revealed the genetic diversity of the *fliC*_H25_ and *fliC*_H28_ gene sequences in *E*. *coli*. Based on allele discrimination of these *fliC*-genes real-time PCR tests were designed for identification of EHEC O145:H25 and O145:H28. The *fliC*_H25_ genes present in O145:H25 were found to be very similar to those present in *E*. *coli* serogroups O2, O100, O165, O172 and O177 pointing to their common evolution but were different from *fliC*_H25_ genes of a multiple number of other *E*. *coli* serotypes. In a similar way, EHEC O145:H28 harbor a characteristic *fliC*_H28_ allele which, apart from EHEC O145:H28, was only found in enteropathogenic (EPEC) O28:H28 strains that shared some common traits with EHEC O145:H28. The real time PCR-assays targeting these *fliC*_H25[O145]_ and *fliC*_H28[O145]_ alleles allow better characterization of EHEC O145:H25 and EHEC O145:H28. Evaluation of these PCR assays in spiked ready-to eat salad samples resulted in specific detection of both types of EHEC O145 strains even when low spiking levels of 1–10 cfu/g were used. Furthermore these PCR assays allowed identification of non-motile *E*. *coli* strains which are serologically not typable for their H-antigens. The combined use of O-antigen genotyping (O145*wzy*) and detection of the respective *fliC*_H25[O145]_ and *fliC*_H28[O145]_ allele types contributes to improve identification and molecular serotyping of *E*. *coli* O145 isolates.

## Introduction

The ability to produce Shiga (Vero) toxins (Stx) was found to be associated with more than 472 different serotypes (O:H types) of *Escherichia coli* [1]. Many of these Shiga toxin-producing *E*. *coli* (STEC) strains are part of the intestinal flora of domestic and wildlife animals and can thus be found in the environment and as contaminants of food. Humans can get infected with STEC by contact with excreting animals or humans, a polluted environment and ingestion of contaminated food [2]. STEC infections in humans can cause diarrheal disease but only a few number of STEC types may cause more severe illness such as hemorrhagic colitis (HC) and hemolytic uremic syndrome (HUS). These latter types of STEC are also designated as enterohemorrhagic *E*. *coli* (EHEC) [3].

EHEC were defined on the basis of the severe clinical picture of the disease they cause, their frequency in outbreaks of disease and the presence of the *eae* (intimin) gene encoded by the LEE (locus of enterocyte effacement), and of non-LEE effector genes located on different genomic islands in the strains [3–5]. At present, five EHEC (“top-five”) serotypes encompassing motile and non-motile strains of O26:H11, O103:H2, O111:H8, O145:H25/H28, and O157:H7 are regarded as most important for public health in the European Union (EU) [6] and in the United States [1–2]. In addition, two other EHEC serotypes, (O45:H2 and O121:H19) were associated with severe disease in humans and therefore included in the panel of EHEC strains (“top-seven”) searched routinely in meat products in the United States [2].

Real-time PCR methods have been developed for specific detection of strains belonging to the most important EHEC types and a number of such methods were described for specific detection of EHEC O145 strains [7–14]. Harmonized real-time PCR procedures developed for the countries of the EU [15] and the U.S. [16] operate in a cascade-like process where first the presence of both *stx* and *eae* genes in the samples is searched. If positive, the presence of genes encoding the O-antigen of the suspected EHEC O-serogroup is investigated. Both procedures became effective in 2012 [2].

Samples from clinical, environmental and food origin frequently contain mixtures of bacteria which may hamper the detection and isolation of suspected EHEC contaminants. Quite often, feces from ruminant animals carry mixtures of Stx-negative *E*. *coli* strains carrying the *eae*-gene (so called atypical EPEC) together with *eae*-negative STEC strains [17]. In case of fecal contamination, food may contain both, atypical EPEC and STEC strains, thus falsely indicating the presence of EHEC when analyzed for the presence of *stx*- and *eae*-genes. Such samples would be subsequently investigated for the presence of one of the seven EHEC O-groups for confirmation [15–16]. However, a positive result for the O-antigen may also be misleading, as many non-EHEC strains occurring in food and clinical samples share the same O-antigen with the top seven EHEC strains [14]. In order to reduce the number of false-positive results in the initial screening of samples for EHEC, the inclusion of more EHEC-specific markers is desirable.

Investigation of flagellar (*fliC*) genes was previously shown to be helpful for detection of EHEC strains with serological cross-reacting H-types [18] and for further characterization of non-motile EHEC strains [19–22]. The *fliC* genes of almost all flagellar antigens in *E*. *coli* have been sequenced [23] and sequence diversity of *fliC* genes was used for identification of EHEC strains such as O157:H7 [24]. Sequence diversity of *fliC* genes was found among *E*. *coli* strains expressing different O-antigens but sharing the same flagellar antigenic type such as H6 and H7 strains [24–25]. *fliC* sequence data derived from different *E*. *coli* H7 strains were used to develop PCR methods for specific detection of the H7 antigen encoded by *E*. *coli* O55 and O157 strains [24].

In this work we describe real-time PCR methods for identification of the *fliC*-genes encoded by EHEC O145:H25 and O145:H28 strains. The combined use of O-antigen genotyping (O145*wzy*) and detection of the respective O145-*fliC* types H25 and H28 improves the identification and molecular O:H serotyping of EHEC O145.

## Materials and Methods

### Bacteria

The isolates were provided from the collections of the National Reference Laboratory for *E*. *coli* (NRL *E*. *coli*) at the Federal Institute for Risk Assessment (BfR) in Berlin, Germany and from the French Agency for Food, Environmental and Occupational Health and Safety (Anses) in Maisons-Alfort, France. *E*. *coli* strains used for the experiments were previously described for their serotypes and their virulence genes [4, 26–30]. The reference strains belonging to *E*. *coli* O-serogroups O1-O181 [30–31] and sixty-six serologically confirmed O145 strains carrying different H-antigens [4, 26–30] were used to test the specificity of O145*wzy* PCR. *E*. *coli* strains expressing the flagellar antigens H25 or H28 were detected by H-serotyping and by nucleotide sequencing of PCR amplified *fliC* products as previously described [19]. Relevant characteristics of *E*. *coli* strains used for nucleotide sequence analysis of their *fliC* genes are listed in Table 1.

**Table 1:** *Escherichia coli* strains used for nucleotide sequencing of *fliC* genes.

Strain	Original number	serotype	*fliC* gene GenBank accession no	STEC Virulence genes	Source and Reference
CB12641	1034–05	O145:H25	LN555738	*stx*_2a_, *eae*,	human feces, [48]
CB12671	2208–08	O145:H25	LN555739	*stx*_2a_, *eae*,	Human feces, [48]
CB12513	2454–01	O145:H28	LN555740	*stx*_1_, *stx*_2a_, *eae*	Human feces, [48]
CB12663	1094–07	O145:H28	LN555741	*stx*_2a_, *eae*	Human feces, [48]
CB13990		O2:H25	LN614384	*stx*_2g_	Cattle feces, Germany, 2012, (this work)
CB9767		O100:H25	LN649616	*eae*	Human feces, [49]
CB10528		O172:H25	LN649617	*stx*_2a_, *eae*	Beef, Germany, 2006, (this work)
CB14727		O177:H25	LN614386	*eae*	Goat milk, Germany, 2013, (this work)
N234		O15:H25	LN649619	none	Cattle feces, [30]
CB11499	NVH-812	O103:H25	LN649618	*eae*	Sheep feces, [50]
CB12546	VTB60	O165:H25	LN614385	*stx*_2a_, *stx*_2c_, *eae*	No data, [51]
CB9651		O28:H28	LN649615	*eae*	Human feces, [49]

### PCR detection and mapping of *E*. *coli* O-antigen and H-antigen genes

**Fig 1:**
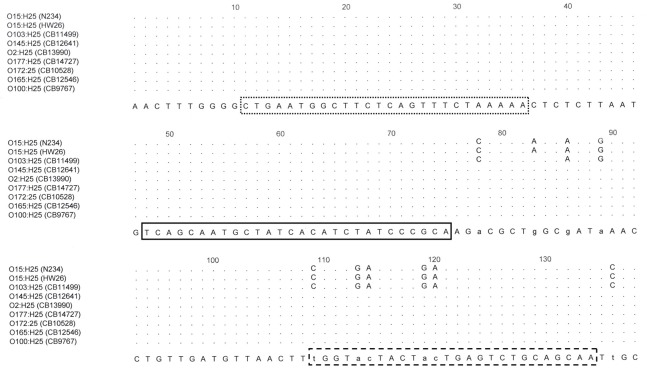
Position of the forward (dotted line) and backward (interrupted line) primers and of the gene probe (solid line) in *fliC*_H25_ sequences for detection of the *fliC*_H25_ genes of EHEC O145:H25 strains.

**Fig 2:**
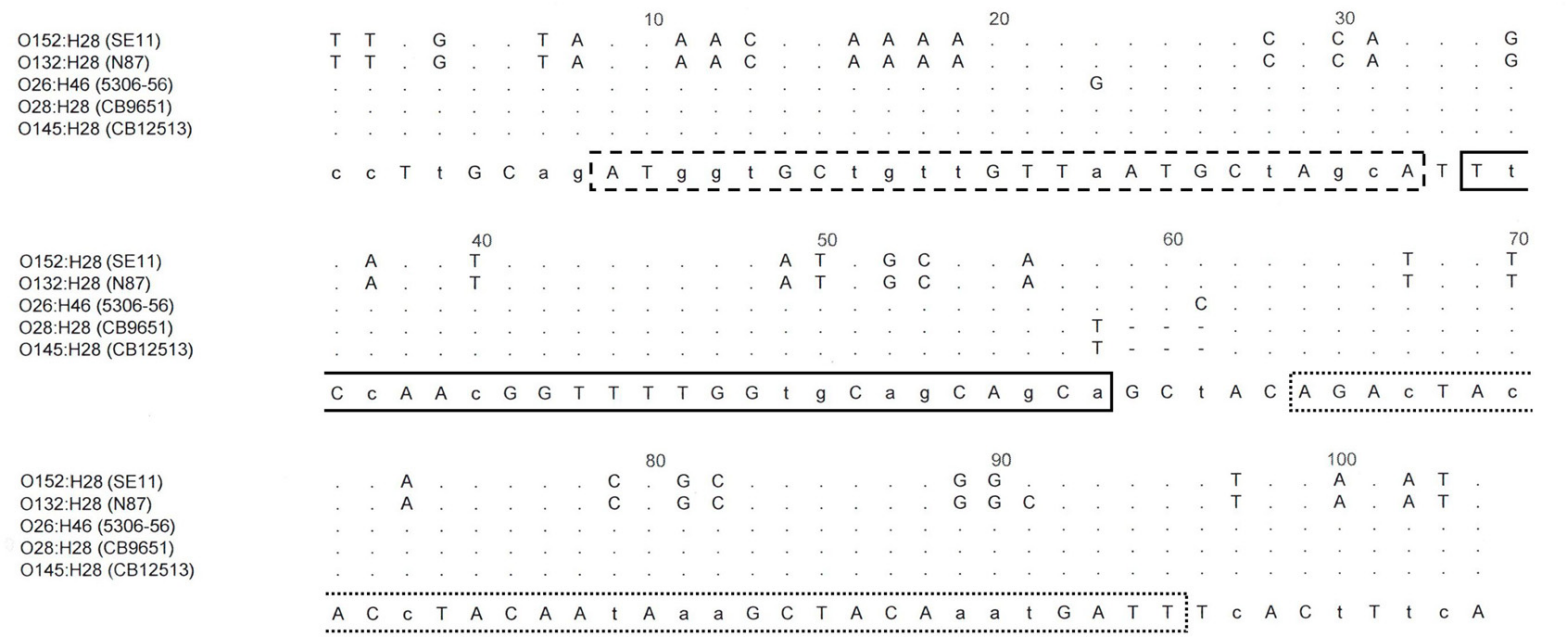
Position of the forward (dotted line) and backward (interrupted line) primers and of the gene probe (solid line) in *fliC*_H28_ sequences for detection of the *fliC*_H28_ genes of EHEC O145:H28.

Mapping of *fliC* gene variants to their respective H-types was performed by PCR and analysis of restriction fragment length polymorphism of *HhaI* digested PCR-products (PCR/RFLP) as previously described [19, 32]. Nucleotide sequence data obtained from different *fliC*_H25_ and *fliC*_H28_ genes were used for designing TaqMan PCR probes and primers for common detection of all genetic variants of *fliC*_H25_ and *fliC*_H28_ genes (Table 2 of this work, [4]) and for identification of O145-specific *fliC*_H25_ (*fliC*_H25[O145]_) and O145-specific *fliC*_H28_ (*fliC*_H28[O145]_) genes (Table 2). The real-time PCR assay specific for the *ihp1* gene of EHEC O145:H28 was described previously [26, 33]. A TaqMan PCR probe and specific primers covering all types of *E*. *coli* O145 strains was deduced from the O145*wzy* (O-antigen polymerase) gene (Table 2). Real-time PCR reactions were set up as singleplex assays. TaqMan PCR probes and primers used in this work were designed with the software Primer Express V3.0 (Applied Biosystems) and are described in Table 2. The position of primers and gene probes for specific detection of O145 *fliC*_H25_ and O145 *fliC*_H28_ genes is indicated in Figs 1 and 2. Real-time PCR amplifications were performed with an ABI 7500 instrument (Applied Biosystems, Foster City, CA, USA) in 25-μl reaction volumes or with a LightCycler 1536 (Roche Diagnostics, Meylan, France) in 1.5 μl reaction volumes according to the recommendations of the suppliers. Briefly, primers and TaqMan probes were used at 300 nM final concentration for the PCR reaction. The following thermal profile was used: 95°C for 1 min followed by 40 cycles of 95°C for 10 s, 60°C for 30s.

**Table 2:** Primers and probes used for real-time PCR assays.

Target gene^a^	Forward, reverse primers and probe sequences (5’- 3’)	Length and location within sequence (5’- 3’)
***fliC***_**H25**_	CACAACATYCTTGATAAAGATGG	607–629 (AY250007)
	AACAGAAGCAGCATAGAAGTC	667–687 (AY250007)
	[6FAM]- GCAACAGCTGATTATGTTGTTCAGTCAGG-[BHQ1]	634–662 (AY200007)
***fliC***_**H25[O145]**_	CTGAATGGCTTCTCAGTTTCTAAAAA	508–533 (LN555738- LN555739)
	TGGTACTACTACTGAGTCTGCAGCAA	606–631 (LN555738- LN555739)
	[6FAM]-TCAGCAATGCTATCACATCTATCCCGCA-[BHQ1]	545–572 (LN555738- LN555739)
***fliC***_**H28**_	AAAACAATGCTGGGACTGTC	[4]
	TTGTAATTACCGTAGATACGGC	[4]
	[6FAM]- AAAAACTGGCATACAACAGGCACACCG-[BHQ1]	[4]
***fliC***_**H28[O145]**_	AGACTACACCTACAATAAAGCTACAAATGATT	750–781 (LN555740- LN555741)
	ATGGTGCTGTTGTTAATGCTAGCA	698–721 (LN555740- LN555741)
	[6FAM]- TTCCAACGGTTTTGGTGCAGCAGCT-[BHQ1]	723–747 (LN555740- LN555741)
***wzy***_**O145**_	CAGCATGGTTATCACTTGGTATGAC	412746–412800 (JASO01000012)
	AATATTTACTCTTGCAAGCATTGGC	412855–412879 (JASO01000012)
	[6FAM]—ACAGTGCCAGCATTCGCTTGCGA—[BHQ1]	412827–412849 (JASO01000012)

### Nucleotide sequencing

The nucleotide sequence of the PCR products were determined by Sanger sequencing [34] and analyzed by the use of the Accelrys DS Gene software package (Accelrys Inc., USA). The nucleotide sequences of the respective products for *fliC* homologs were determined and have been submitted to European Nucleotide Archive (ENA). Accession numbers are listed in Table 1.

### Detection of EHEC O145:H25 and EHEC O145:H28 from spiked ready-to eat-salad samples by real-time PCR

A lot of ready-to-eat, pre-cut salad was purchased at wholesale and spiked with high (100–1000 cfu/ gram) and low (1–10 cfu/ gram) quantities of EHEC O145:H25 (CB12641) and EHEC O145:H28 (CB12513) strains (Table 1). Salad samples spiked with non-O145 EHEC (CB14655, O121:H19, *stx*_2a_, *eae*), STEC (CB15589, O104:H7, *stx*_1c_) and EPEC (CB12054, O157:H45, *eae*) strains as well a native, unspiked salad sample served as negative controls. Determination of the bacterial microflora present in native samples was performed on Standard I agar (total mesophilic counts) and Violet Red Bile (VRB) agar (*Enterobacteriaceae*) as described previously [35]. Spiking of salad samples and preparation of DNA from enrichment cultures of bacteria was performed as described previously [35]. Spiked and native salad samples were kept for 24h at 6°C before further processing. Primers and probes for real-time PCR detection of *stx*_1_, *stx*_2_, *eae* and O145*ihp1* genes were previously described [26, 35]. Primers and probes for detection of O145*wzy*, *fliC*_H25_ and *fliC*_H28_ genes are listed in Table 2.

## Results

### Development of an *E*. *coli* O145 serogroup specific real-time PCR-assay

The O145*wzy* PCR has been tested for its specificity on reference strains belonging to *E*. *coli* O-serogroups O1-O186 and was found to react only with the O145 reference strain (E1385, O145:NM) [30]. Also, the O145*wzy* PCR was positively tested on a collection of sixty-six serologically confirmed O145 strains carrying different H-antigens (Table 3). As the O145*wzy* PCR proved to be specific it was used to confirm results obtained by O-serotyping and for screening for the presence of O145 O-antigen genes in serologically Orough:H25 and Orough:H28 strains (see below).

**Table 3:** Specificity of the O145*wzy* real-time PCR within strains belonging to serogroup O145.

Serotype^a^	number of strains	CT values^b^
O145:H25	6	18.7–23.3
Orough:H25	5	14.1–20.3
O145:H28^c^	36	15.7–23.8
Orough:H28^c^	2	14.1–21.9
O145:H34	9	16.7–22.1
O145:H1	2	21.4–21.8
O145:H2	2	22.3–23.1
O145:H19	1	21.3
O145:Hnt	3	16.7–22.3

a) some of these strains were non-motile and the *fliC*-genotype was detected by nucleotide sequencing of *fliC* PCR products. Hnt = H-Antigen not typable

b) Range of real time PCR cycle thresholds

c) Only these strains reacted positive in the O145 *ihp1* gene PCR

### Characteristic nucleotide sequences associated with *fliC* genes of EHEC O145:H25 and O145:H28 strains

The *fliC* sequences obtained from *E*. *coli* O145:H25 and O145:H28 strains revealed characteristic differences when compared to non-O145:H25 and non-O145:H28 serotypes. For further examination, the complete *fliC* nucleotide sequences of both EHEC O145:H25 and EHEC O145:H28 strains were determined.

The H25 flagellin encoding genes derived from two *E*. *coli* O145:H25 strains CB12641 (GenBank Accession LN555738) and CB12671 (GenBank Accession LN555739) had each a length of 1332 bp and were identical between both strains. BLAST search revealed 100% and 99% sequence identity with partial *fliC* sequences of O145 strains 4392/97 (GenBank Accession AJ566340.1) and 2839/98 (GenBank Accession AJ566341.1) respectively [22] and 98% identity with the partial *fliC*_H25_ sequence (GenBank Accession AY250007) of the O15:H25 type reference strain HW26 [23]. Nucleotide sequences identical to those obtained from CB12641 and CB12671 were found in whole genome sequencing (WGS)-contigs deposited at GenBank (Accession numbers JHMZ01000105.1, JHHD01000016.1, JASO01000012.1, AIAX01000332.1 and AIAQ01000055.1). Among the WGS-contigs deposited at GenBank two are of unknown serotype, one is O145:H25 and one is O177:H25.

The complete *fliC* sequences of two EHEC O145:H28 strains, CB12513 (GenBank Accession LN555740) and CB12663 (GenBank Accession LN555741) were determined. These were identical to each other with a length of the flagellin encoding region of 1743bp (580 aa). The *fliC* sequences of CB12513 and CB12663 were identical to four *fliC* sequences (all 1743bp) derived from O145:H28 strains (GenBank Accession CP007136.1, CP007133.1, CP006027.1 and CP006262.1) [36–37]. In contrast, the *fliC*_H28_ sequences of O145:H28 strains showed only 92% similarity (1617/1752 bp) to the complete *fliC*_H28_ gene of the H28 type reference strain HW30 (O132:H28) which has a length of 1752bp [23] (GenBank Accession AY250010) and 92.5% identity to the *fliC*_H28_ sequence of strain SE11 (O152:H28) (GenBank Accession AP009240.1) which has a total length of 1740 bp [38] (GenBank Accession AP009240.1).

The differences found in the *fliC*_H25_ and *fliC*_H28_ genes of EHEC O145 strains compared to non-O145 strains carrying the same flagellar antigens prompted us to develop real-time PCR assays to improve the identification of these clinically important EHEC O145 types.

### Development of a *fliC* real-time PCR for detection of EHEC O145:H25 strains

Primers and TaqMan PCR probe covering a segment specific for the EHEC *fliC*_H25[O145]_ gene were tested on a collection of 68 *E*. *coli* strains which carried the *fliC*_H25_ gene as tested by H-serotyping or nucleotide sequence typing of their *fliC* genes and a generic *fliC*_H25_ PCR targeting common regions of the *fliC*_H25_ genes. The 68 strains comprised 26 different O-serogroups, as well as O-untypable (ONT) and Orough (Or) strains (Table 4).

**Table 4:** Reaction of the *fliC*_H25[O145]_ Real Time PCR on different *E*. *coli* H25 antigen type strains.

Serotype^a^	numbers of strains	motility^b^	CT values^c^
O2:H25	5	5	20.5–26.1
O8:H25	1	1	0
O11:H25	1	0	0
O15:H25	2	1	0
O21:H25	2	2	0
O23:H25	1	1	0
O36:H25	1	1	0
O45:H25	1	1	0
O66:H25	1	1	0
O71:H25	1	1	0
O88:H25	1	1	0
O98:H25	1	0	0
O100:H25	1	0	19.2
O103:H25	4	2	0
O111:H25	2	1	0
O119:H25	2	2	0
O123:H25	1	0	0
O145:H25	6	4	18.7–20.6
Orough [O145*wzy*]:H25^d^	5	5	20.2–22.7
O147:H25	1	1	0
O153:H25	4	4	0
O156:H25	1	1	0
O165:H25	3	2	19.1–25.4
O171:H25	1	1	0
O172:H25	1	0	22.5
O177:H25	7	2	18.7–23.8
O182:H25	2	1	0
ONT:H25	6	4	0
Orough:H25	3	3	0

a) All 68 strains reacted positive with the generic *fliC*_H25_ PCR described in Table 2 which targets common regions of the *fliC*_H25_ genes.

b) Number of motile strains detectable by H-serotyping.

c) Range of real time PCR cycle thresholds

d) these O-rough strains were positive for the *E*. *coli* O145 specific *wzy*-gene (O-antigen polymerase).

The *fliC*_H25[O145]_ PCR was positive with all six O145:H25 strains, with one Or:H25 strain carrying the O145*wzy* gene, as well as with O2:H25 (n = 5), O100:H25 (n = 1), O165:H25 (n = 3), O172:H25 (n = 1) and O177:H25 (n = 7) strains. The remaining 28 *E*. *coli* H25 strains divided into 20 other O-serogroups as well as three Orough and four ONT strains were all negative in the *fliC*_H25[O145]_ gene PCR (Table 4).

**Fig 3:**
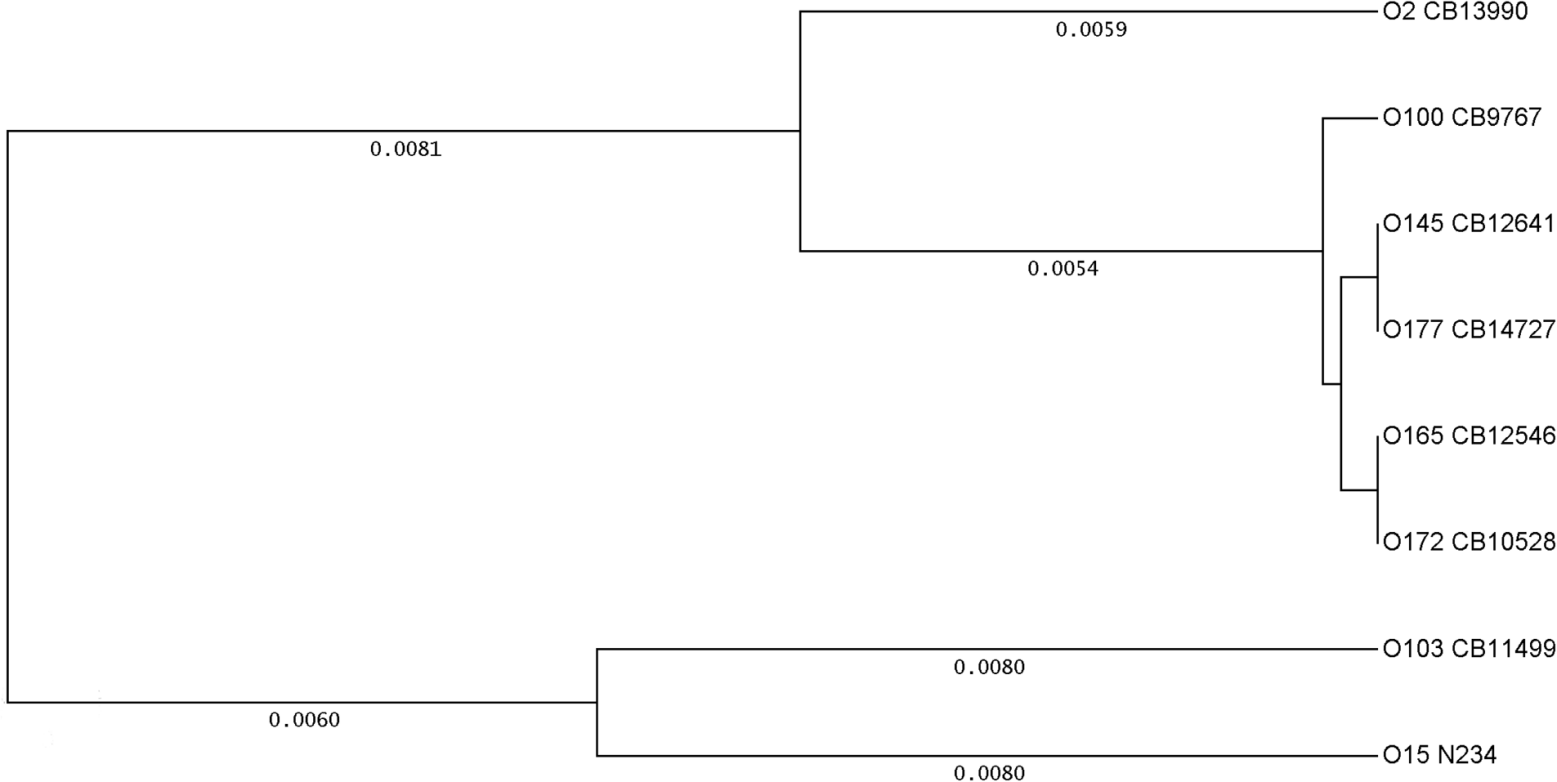
Genetic relationship between *fliC*_*H25*_ genes in different strains and serotypes of *E*. *coli*. Cluster analysis of the *fliC*_**H25**_ genes present in *E*. *coli* strains belonging to the following serotypes: O145:H25: CB12641 (GenBank Accession LN555738), O177:H25: CB14727 (LN614386), O100:H25: CB9767 (LN649616), O165:H25: CB12546 (LN614385), O172:H25: CB10528 (LN649617), O2:H25: CB13990 (LN614384), O103:H25: CB11499 (LN649618) and O15:H25: N234 (LN649619). The O145:H25 strain CB12671 (LN555739) has a *fliC* sequence identical to that of CB12513 (LN555740) and is therefore not shown in the figure. UPGMA was used as tree building mode and the distances calculated according to Tajima and Nei [47].

Since *fliC*_H25[O145]_ PCR showed cross-reactions with some non-O145:H25 strains we determined the *fliC*_H25_ sequence of each one representative strain per serogroup O2:H25 (CB13990), O100:H25 (CB9767), O165:H25 (CB12546), O172:H25 (CB10528) O177:H25 (CB14727). The flagellin encoding gene derived from these strains had each a length of 1332 bp (443 aa). Homology between the *fliC*_H25_ genes of *E*. *coli* O2, O100, O145, O165, O172 and O177 was found in the target region of the *fliC*_H25[O145]_ real-time PCR (508–631 bp, Table 2). The *fliC* sequences derived from these *fliC*_H25[O145]_ gene PCR-reacting strains were compared to those of representative non-reacting strains such as N234 (O15:H25) and CB11499 (O103:H25). A cluster analysis of the *fliC*_H25_ coding sequences is presented in Fig 3. A cluster of genetically closely related strains is formed by *fliC*_H25[O145]_ gene PCR reacting strains (O2, O100, O103, O145, O165, O172 and O177). The *fliC*_H25_ sequences of PCR non-reacting strains N234 and CB11499 were found genetically more distant (Fig 3).

In detail, the *fliC* sequence of the *E*. *coli* O177:H25 strain was identical with those of investigated EHEC O145:H25 strains. The *fliC* sequences of the O165:H25 and the O172:H25 strains differed from O145:H25 by one nucleotide exchange at the same position within the sequence (C/T, position 1134). The *fliC* sequence of the O100:H25 strain showed one nucleotide exchange at another position (G/A, position 110). These nucleotide exchanges had no effect on the derived amino-acid (aa) sequence (S1 Fig). On the other hand, changes in the amino acid composition of the H25 flagellins compared to that of O145:H25 were observed for the genetically more distant strains O2:H25 (4 aa), O15:H25 (7 aa) and O103:H25 (8aa) (S1 Fig). The specificity of the *fliC*_H25[O145]_ PCR assay was further tested on a panel of strains including reference strains encompassing H-types H1 to H56. None of these strains gave a positive result with the *fliC*_H25[O145]_ PCR (data not shown).

### Development of a *fliC* real-time PCR for detection of EHEC O145:H28 strains

The real-time PCR developed from the *fliC*_H28[O145]_ sequence was tested on 70 strains identified as H28 positive by serotyping or nucleotide sequence typing of *fliC* genes and by a generic *fliC*_H28_ PCR targeting common regions of the *fliC*_H28_ genes. The strains belonged to 14 different O groups, as well as to ONT and Orough strains. The results are summarized in Table 5. The *fliC*_H28[O145]_ PCR reacted with all 35 O145:H28 and two Orough:H28 strains which were positive for the O145*wzy* gene. Except for four O28:H28 strains, no other strain expressing flagellar type H28 reacted in the *fliC*_H28[O145]_ PCR. In order to explore the nature of the observed reaction with O28:H28 strains we determined the nucleotide sequence of the *fliC* gene of a representative strain (CB9651, O28:H28, GenBank LN649615). The sequence had the same length and was identical to that found with O145:H28 strains.

**Table 5:** Reaction of the *fliC*_H28[O145]_ real time PCR on different *E*. *coli* H28 antigen type strains.

Serotype^a^	numbers of strains	motility^b^	CT values^c^
O8:H28	1	1	0
O21:H28	1	1	0
O28:H28	4	1	17.4–18.4
O68:H28	1	1	0
O74:H28	1	1	0
O91:H28	1	1	0
O110:H28	2	2	0
O116:H28	1	0	0
O132:H28	2	2	0
O145:H28	36	8	14.8–23.5
Orough[O145*wzy*]:H28^d^	2	2	21.1–23.5
O146:H28	1	1	0
O166:H28	1	1	0
O174:H28	1	1	0
O185:H28	2	2	0
ONT:H28	4	3	0
Orough:H28	9	8	0

a) All 70 strains reacted positive with the generic *fliC*_H28_ PCR described in Table 2 which targets common regions of the *fliC*_H28_ genes.

b) Number of motile strains detectable by H-serotyping

c) Range of real time PCR cycle thresholds

d) Orough strains that were positive for the *E*. *coli* O145 *wzy*-gene.

**Fig 4:**
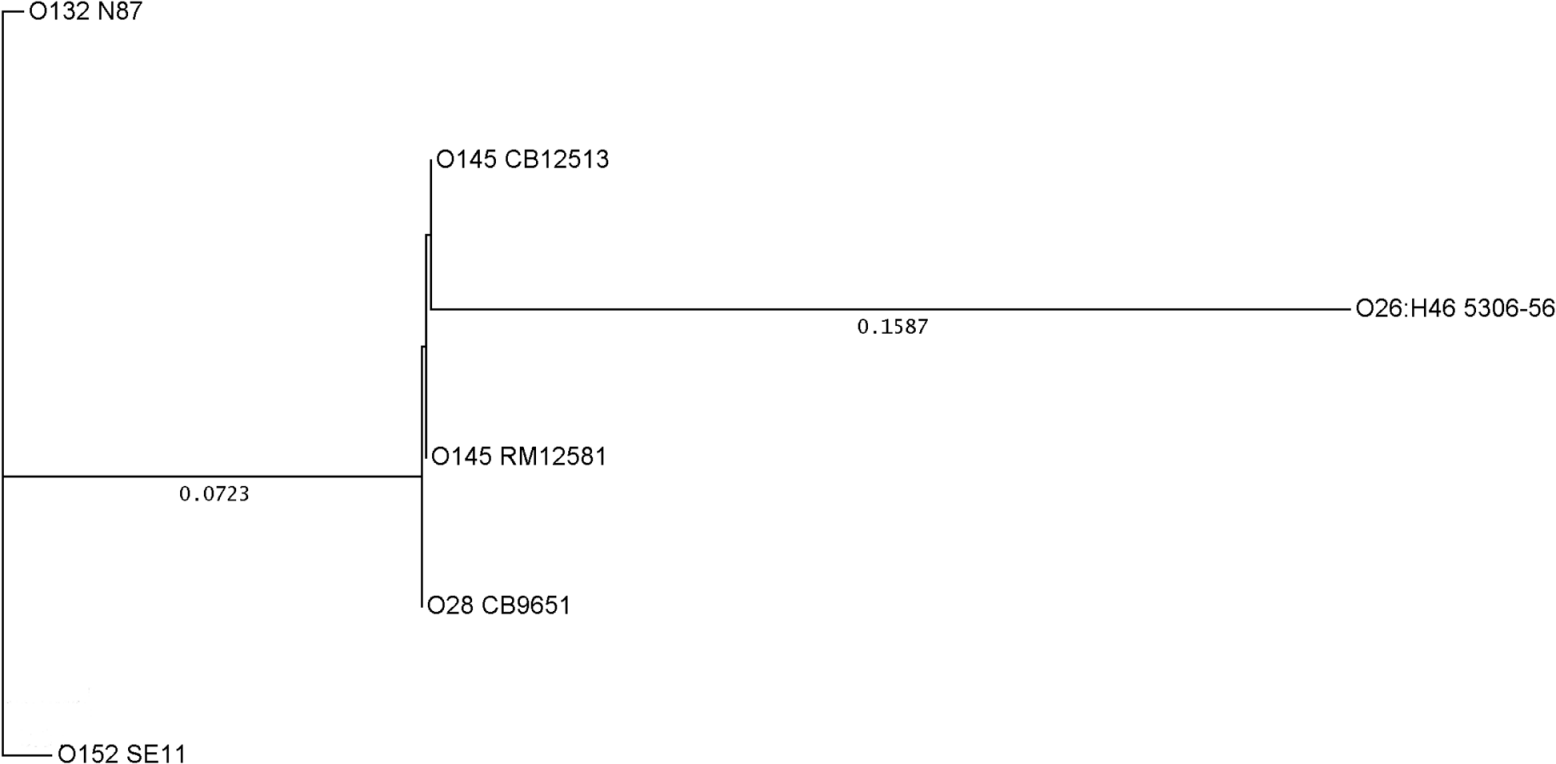
Genetic relationship between *fliC*_H28_ genes in different strains and serotypes of *E*. *coli*. Cluster analysis of the *fliC*_**H28**_ genes present in *E*. *coli* strains belonging to the following serotypes: O145:H28: CB12513 (GenBank Accession LN555740), RM12581 (CP007136.1), O28:H28: CB9651 (LN649615), O132:H28: HW30 (AY250010) and O152:H28 (AP009240.1). The O145:H28 strain CB12663 (LN555741) has a *fliC* sequence identical to that of CB12513 (LN555740) and is therefore not shown in the figure. The sequence of the O26:H46 strain 5306–56 (AY250024) was compared to those of *E*. *coli* H28 strains because of their similarity in the target region for the *fliC*_**H28[O145]**_ PCR. UPGMA was used as tree building mode and the distances calculated according to Tajima and Nei [47].

A cluster analysis of *fliC*_H28_ sequences derived from *fliC*_H28[O145]_ PCR reacting (*E*. *coli* O145:H28 and O28:H28) and non-reacting (*E*. *coli* O132:H28 and O152:H28) strains revealed two genetic clusters of strains that were distinguishable by the *fliC*_H28[O145]_ PCR (Fig 4). None of the *E*. *coli* H-type reference strains H1-H56 reacted in the *fliC*_H28[O145]_ PCR except for strain 5306–56 (O26:H46) which gave a positive reaction with a cycle threshold (CT) value of 21.3. The reaction was supposed to be *fliC* specific as another H46 strain from our collection (CB13742, Or:H46) reacted equally well (CT value of 21.9) in the PCR. By comparing the corresponding regions in the EHEC *fliC*_H28[O145]_ genes and the *fliC*_H46_ gene of strain 5306–56 (GenBank AY250024, 1719bp), only small differences were found (one mismatch in the backward primer and one mismatch in the probe sequence). The sequence similarity in the target region could explain the PCR cross-reaction with flagellar type H46 strains. Comparison of the *fliC* gene sequences of five *E*. *coli* H28 strains belonging to serogroups O145 (CB12513 and RM12581), O28 (CB9651), O132 (N87) and O152 (SE11) with that of strain 5306–56 (O26:H46) revealed that the latter is genetically more distant from all these (Fig 4).

### Evaluation of the O145:H25 and EHEC O145:H28 real-time PCR detection systems on artificially contaminated mixed salad samples

Two random 25-g portions of the salad used for spiking experiments were analyzed for their bacterial microflora. The total mesophilic counts on Standard I agar (25°C) were 2.06–3.26 x 10^6^ cfu/g. Lactose-negative and lactose-positive *Enterobacteriaceae* were detected in quantities of 9.0 x 10^4^–1.89x10^5^ cfu/g after growth on VRB agar for 22h at 37°C. Portions of salad (25g) were spiked with high (100–1000 cfu/g) and low (1–10 cfu/g) quantities of the *E*. *coli* strains described in Table 6. DNA preparations obtained from enrichment cultures of eight artificially contaminated mixed salad samples and one native control were investigated by real-time PCR for EHEC and EHEC O145 specific markers *stx*1, *stx*2, *eae*, O145_ihp1_, O145_wzy_, *fliC*_H25[O145]_ and *fliC*_H28[O145]._ The results are summarized in Table 6. All tested strains were confirmed for the presence of their virulence genes. The EHEC O145:H25 and O145:H28 strains could be specifically detected from contaminated salad using the O145_wzy_, *fliC*_H25[O145]_ and *fliC*_H28[O145]_ detection systems described in this work.

**Table 6:** Detection of O145:H25 and O145:H28 strains from enrichment cultures of artificially contaminated salad samples.

				Real time PCR CT values^a^						
Salad sample	Strain^b^	Serotype	cfu/g salad^c^	*stx1*	*stx2*	*eae*	O145*ihp1*	O145*wzy*	*fliC*_H25[O145]_	*fliC*_H28[O145]_
1	CB12054	O157:H45	100–1000	0	0	17.5	0	0	0	0
2	CB15589	O104:H7	100–100	18.4	0	0	0	0	0	0
3	n.a.	n.a.	0	0	0	0	0	0	0	0
4	CB14655	O121:H19	1–10	0	20.9	20.7	0	0	0	0
5	CB12641	O145:H25	100–1000	0	23.0	23.0	0	24.4	24.9	0
6	CB12513	O145:H28	1–10	20.3	20.0	19.9	21.7	21.1	0	20.4
7	CB14655	O121:H19	100–1000	0	18.9	18.9	0	0	0	0
8	CB12641	O145:H25	1–10	0	29.0	28.2	0	29.8	29.1	0
9	CB12513	O145:H28	100–1000	19.1	18.8	13.3/22.8	18.1	19.2	0	19,.4

a) Summary of real time PCR cycle thresholds from two enrichment cultures.

b) strain taken for inoculation,

c) inoculation quantity.

## Discussion

Shiga-toxin producing *E*. *coli* O145 are known as the causative agents of severe illness such as hemorrhagic colitis (HC) and hemolytic uremic syndrome (HUS) worldwide. EHEC O145 encompass motile and non-motile strains of serotypes O145:H25 and O145:H28. While EHEC O145:H28 strains have been described for many years, EHEC O145:H25 were identified more recently as a second important type among serogroup EHEC O145 strains and were isolated from human patients [22, 39–40].

A number of different real-time PCR assays were developed for detecting EHEC O145 strains. Some of these methods are based on the presence of the *ihp1* gene [8, 9, 14, 26, 41] which is specific for O145:H28 strains [26]. However, the O145 *ihp1* PCR-assay does not react with O145 strains carrying other H-antigens [26] including EHEC O145:H25 strains (Table 3, this work). Other real-time PCR methods have used *E*. *coli* O145 O-antigen specific *wzx* or *wzy* sequences as targets for specific detection of these strains ([7, 10, 12], this study). In contrast to the *ihp1*-based PCR which detects only O145:H28 and shows cross-reactions with some strains belonging to other O-serogroups [26], the O145_*wzy*_ and O145_*wzx*_ PCR assays are potentially suitable to detect all variants of serogroup O145 strains including both EHEC types O145:H25 and O145:H28. This was demonstrated using the O145_*wzy*_ PCR, which was specific, giving no cross reactions with strains belonging to O-groups other than O145 (this work). Many of the so far described real-time PCR assays use combinations of different genes for a more specific detection of EHEC O145 and other EHEC strains. For example, assays targeting both *eae*-γ1 (intimin) and *fliC*_H28_ sequences were developed for specific detection of EHEC O145:H28 strains [13, 14]. However, these assays are not suitable for specific detection of O145:H25 strains which carry *fliC*_H25_ and the *eae*-β genes [22].

In this study we have employed the sequence diversity of *fliC*_H25_ and *fliC*_H28_ genes for developing diagnostic assays for detection of both EHEC O145:H25 and EHEC O145:H28 strains. Molecular typing of *fliC* genes allows further characterization of non-motile (NM) *E*. *coli* strains which are serologically not-typable for their H-antigens [18–19, 21–22]. This was found to be important for characterization of EHEC O145:H28 strains, which were represented by a high number of non-motile variants in our study (Tables 4 and 5, this work).

*FliC* genotyping was also found useful to discriminate clearly between serologically cross-reacting flagellar antigens H8 and H40 and genetic subtypes of flagellar antigen H8 were identified and associated with EHEC O111 and STEC strains belonging to other O-groups [18]. Analysis of the *fliC* genes encoding the H7 antigen in *E*. *coli* strains belonging to different O-groups revealed ten different alleles and PCRs specific for the *fliC* gene carried by EHEC O157:H7 and O157:HNM were developed for DNA-based typing [24–25].

Isolates of classical EHEC strains belonging to serogroups O26, O111, O145 and O157 are frequently non–motile and their H-type can thus only be determined by molecular characterization [19, 22, 25, 42–44]. Moreover, EHEC belonging to serogroup O145 were found to split into two serotypes, O145:H25 and O145:H28 that possess different alleles of the *eae*-gene [22]. Flagellar-types H25 and H28 are widely spread among *E*. *coli* strains belonging to different O-groups and pathotypes ([30, 40, 44], this work).

In order to better characterize EHEC O145:H25 and EHEC O145:H28 strains we have developed real time PCR-assays targeting the *fliC* alleles present in these strains. The *fliC*_H25_ genes present in O145:H25 were found very similar to those of some other STEC and EPEC strains such as O2, O100, O165, O172 and O177 pointing to their common evolution but were different from *fliC*_H25_ genes of a multiple number of *E*. *coli* serotypes including STEC, EPEC and apathogenic strains. The high similarity between the EHEC O145 and *E*. *coli* O2, O100, O165, O172 and O177 *fliC*_H25_ genes (between 100% and 98.87% identity) prevents the design of a real-time PCR assay that would allow a complete discrimination.

In a similar way, a specific real-time PCR assay for detection of O145:H28 targeting its *fliC*_H28_ allele was developed. Apart from EHEC O145:H28 this variant was only found in EPEC O28:H28 strains which shared some common traits with EHEC O145:H28 (*eae*-gamma, *espK*, *espV*, *espN* and *espM1*, the OI-57 markers Z2096, Z2098, Z2099 and Z2121, and the EHEC O145:H28-specific CRISPR marker SP_O145) [4, 27, 45–46]. Only one serotype (H46) was found to cross-react with the newly designed real-time PCR assay. This could lead to misidentification of O145:H46 strain as O145:H28. However, O145:H46 was not previously found in *stx*-positive strains.

Evaluation of the specific real-time PCR assays for EHEC O145:H25 and O145:H28 strains with artificially contaminated ready-to eat salad samples resulted in specific detection of both types of EHEC O145 strains even when inoculated in low quantities (1–10 CFU/g) after overnight enrichment. The presence of a dense natural microflora (> 10^6^ CFU/g salad) did not affect detection of the inoculated EHEC strains.

To our knowledge, the genetic diversity of *fliC*_H28_ and *fliC*_H25_ has not been previously studied in *E*. *coli*. The *fliC*_H28_ and *fliC*_H25_ alleles described in this work contribute to a better characterization of the flagellar antigens of *E*. *coli*. Also, the combination of a new O145_*wzy*_ PCR with the *fliC*_H25[O145]_ and *fliC*_H28[O145]_ PCR assays improves the identification and characterization of EHEC O145 strains.

## Supporting Information

S1 Fig Alignment of the H28 and H46 flagellin amino-acid sequences. (PDF)
