# Supplementary material for: Sequence Variations in the Flagellar Antigen Genes fliC H25 and fliC H28 of Escherichia coli and Their Use in Identification and Characterization of Enterohemorrhagic E. coli (EHEC) O145:H25 and O145:H28
Source: PLoS One. 2015 May 22;10(5):e0126749. doi: 10.1371/journal.pone.0126749 (PMC4441469; doi:10.1371/journal.pone.0126749)
Supplement: S1 Fig — (PDF) [file pone.0126749.s001.pdf]

Alignment Name: Translation of H28 and H46 strains.aaa_bsml
Length: 585

										10										20										30										40						
Translation of O145 CB12513	M	A	Q	V	I	N	T	N	S	L	S	L	I	T	Q	N	N	I	N	K	N	Q	S	A	L	S	S	S	I	E	R	L	S	S	G	L	R	I	N	S	A	K	D	D	A	A
Translation of O145 RM12581	M	A	Q	V	I	N	T	N	S	L	S	L	I	T	Q	N	N	I	N	K	N	Q	S	A	L	S	S	S	I	E	R	L	S	S	G	L	R	I	N	S	A	K	D	D	A	A
Translation of O28 CB9651	M	A	Q	V	I	N	T	N	S	L	S	L	I	T	Q	N	N	I	N	K	N	Q	S	A	L	S	S	S	I	E	R	L	S	S	G	L	R	I	N	S	A	K	D	D	A	A
Translation of O152 SE11	M	A	Q	V	I	N	T	N	S	L	S	L	I	T	Q	N	N	I	N	K	N	Q	S	A	L	S	S	S	I	E	R	L	S	S	G	L	R	I	N	S	A	K	D	D	A	A
Translation of O132 N87	M	A	Q	V	I	N	T	N	S	L	S	L	I	T	Q	N	N	I	N	K	N	Q	S	A	L	S	S	S	I	E	R	L	S	S	G	L	R	I	N	S	A	K	D	D	A	A
Translation of O26:H46 5306-56	M	A	Q	V	I	N	T	N	S	L	S	L	I	T	Q	N	N	I	N	K	N	Q	S	A	L	S	S	S	I	E	R	L	S	S	G	L	R	I	N	S	A	K	D	D	A	A
	M	A	Q	V	I	N	T	N	S	L	S	L	I	T	Q	N	N	I	N	K	N	Q	S	A	L	S	S	S	I	E	R	L	S	S	G	L	R	I	N	S	A	K	D	D	A	A

				50										60										70										80										90		
Translation of O145 CB12513	G	Q	A	I	A	N	R	F	T	S	N	I	K	G	L	T	Q	A	A	R	N	A	N	D	G	I	S	V	A	Q	T	T	E	G	A	L	S	E	I	N	N	N	L	Q	R	I
Translation of O145 RM12581	G	Q	A	I	A	N	R	F	T	S	N	I	K	G	L	T	Q	A	A	R	N	A	N	D	G	I	S	V	A	Q	T	T	E	G	A	L	S	E	I	N	N	N	L	Q	R	I
Translation of O28 CB9651	G	Q	A	I	A	N	R	F	T	S	N	I	K	G	L	T	Q	A	A	R	N	A	N	D	G	I	S	V	A	Q	T	T	E	G	A	L	S	E	I	N	N	N	L	Q	R	I
Translation of O152 SE11	G	Q	A	I	A	N	R	F	T	S	N	I	K	G	L	T	Q	A	A	R	N	A	N	D	G	I	S	V	A	Q	T	T	E	G	A	L	S	E	I	N	N	N	L	Q	R	I
Translation of O132 N87	G	Q	A	I	A	N	R	F	T	S	N	I	K	G	L	T	Q	A	A	R	N	A	N	D	G	I	S	V	A	Q	T	T	E	G	A	L	S	E	I	N	N	N	L	Q	R	I
Translation of O26:H46 5306-56	G	Q	A	I	A	N	R	F	T	S	N	I	K	G	L	T	Q	A	A	R	N	A	N	D	G	I	S	V	A	Q	T	T	E	G	A	L	S	E	I	N	N	N	L	Q	R	V
	G	Q	A	I	A	N	R	F	T	S	N	I	K	G	L	T	Q	A	A	R	N	A	N	D	G	I	S	V	A	Q	T	T	E	G	A	L	S	E	I	N	N	N	L	Q	R	i

								100										110										120										130								
Translation of O145 CB12513	R	E	L	T	V	Q	A	S	T	G	T	N	S	D	S	D	L	D	S	I	Q	D	E	I	K	S	R	L	D	E	I	D	R	V	S	G	Q	T	Q	F	N	G	V	N	V	L
Translation of O145 RM12581	R	E	L	T	V	Q	A	S	T	G	T	N	S	D	S	D	L	D	S	I	Q	D	E	I	K	S	R	L	D	E	I	D	R	V	S	G	Q	T	Q	F	N	G	V	N	V	L
Translation of O28 CB9651	R	E	L	T	V	Q	A	S	T	G	T	N	S	D	S	D	L	D	S	I	Q	D	E	I	K	S	R	L	D	E	I	D	R	V	S	G	Q	T	Q	F	N	G	V	N	V	L
Translation of O152 SE11	R	E	L	T	V	Q	A	S	T	G	T	N	S	D	S	D	L	D	S	I	Q	D	E	I	K	S	R	L	D	E	I	D	R	V	S	G	Q	T	Q	F	N	G	V	N	V	L
Translation of O132 N87	R	E	L	T	V	Q	A	S	T	G	T	N	S	D	S	D	L	D	S	I	Q	D	E	I	K	S	R	L	D	E	I	D	R	V	S	G	Q	T	Q	F	N	G	V	N	V	L
Translation of O26:H46 5306-56	R	E	L	T	V	Q	A	T	T	G	T	N	S	Q	S	D	L	D	S	I	Q	D	E	I	K	S	R	L	D	E	I	D	R	V	S	G	Q	T	Q	F	N	G	V	N	V	L
	R	E	L	T	V	Q	A	s	T	G	T	N	S	d	S	D	L	D	S	I	Q	D	E	I	K	S	R	L	D	E	I	D	R	V	S	G	Q	T	Q	F	N	G	V	N	V	L

Alignment Name: Translation of H28 and H46 strains.aaa_bsml
Length: 585

		140										150										160										170										180				
Translation of O145 CB12513	A	K	D	G	S	M	K	I	Q	V	G	A	N	D	G	Q	T	I	T	I	D	L	K	K	I	D	S	D	T	L	G	L	S	G	F	N	V	N	G	G	G	A	V	A	N	T
Translation of O145 RM12581	A	K	D	G	S	M	K	I	Q	V	G	A	N	D	G	Q	T	I	T	I	D	L	K	K	I	D	S	D	T	L	G	L	S	G	F	N	V	N	G	G	G	A	V	A	N	T
Translation of O28 CB9651	A	K	D	G	S	M	K	I	Q	V	G	A	N	D	G	Q	T	I	T	I	D	L	K	K	I	D	S	D	T	L	G	L	S	G	F	N	V	N	G	G	G	A	V	A	N	T
Translation of O152 SE11	A	K	D	G	S	M	K	I	Q	V	G	A	N	D	G	Q	T	I	T	I	D	L	K	K	I	D	S	D	T	L	G	L	S	G	F	N	V	N	G	G	G	A	V	A	N	T
Translation of O132 N87	A	K	D	G	S	M	K	I	Q	V	G	A	N	D	G	Q	T	I	T	I	D	L	K	K	I	D	S	D	T	L	G	L	S	G	F	N	V	N	G	S	G	A	V	A	N	T
Translation of O26:H46 5306-56	A	K	D	G	S	M	K	I	Q	V	G	A	N	D	G	Q	T	I	T	I	D	L	K	K	I	D	S	S	T	L	K	L	T	G	F	N	V	N	G	S	G	S	V	A	N	T
	A	K	D	G	S	M	K	I	Q	V	G	A	N	D	G	Q	T	I	T	I	D	L	K	K	I	D	S	d	T	L	g	L	s	G	F	N	V	N	G	g	G	a	V	A	N	T

						190										200										210										220										230
Translation of O145 CB12513	A	A	T	K	D	E	L	A	A	A	A	A	A	A	A	G	T	T	P	A	V	G	T	D	G	V	T	K	Y	T	V	D	A	G	L	N	K	A	T	A	A	N	V	F	A	N
Translation of O145 RM12581	A	A	T	K	D	E	L	A	A	A	A	A	A	A	A	G	T	T	P	A	V	G	T	D	G	V	T	K	Y	T	V	D	A	G	L	N	K	A	T	A	A	N	V	F	A	N
Translation of O28 CB9651	A	A	T	K	D	E	L	A	A	A	A	A	A	A	A	G	T	T	P	A	V	G	T	D	G	V	T	K	Y	T	V	D	A	G	L	N	K	A	T	A	A	N	V	F	A	N
Translation of O152 SE11	A	A	T	K	S	D	L	A	A	A	Q	L	L	A	P	G	T	A	D	A	N	-	-	-	G	T	V	T	Y	T	V	S	A	G	L	K	T	S	T	A	A	D	V	I	A	S
Translation of O132 N87	A	A	T	K	S	D	L	A	A	A	Q	L	L	A	P	G	T	A	D	A	N	-	-	-	G	T	V	T	Y	T	V	G	A	G	L	K	T	S	T	A	A	D	V	I	A	S
Translation of O26:H46 5306-56	A	A	T	K	D	E	L	A	A	A	A	A	-	A	A	G	T	T	P	A	V	G	T	D	G	V	T	K	Y	T	V	D	A	G	L	N	K	A	T	A	A	N	V	F	A	N
	A	A	T	K	d	e	L	A	A	A	a	a	a	A	a	G	T	t	p	A	v	G	T	D	G	v	t	k	Y	T	V	d	A	G	L	n	k	a	T	A	A	n	V	f	A	n

										240										250										260										270						
Translation of O145 CB12513	L	A	D	G	A	V	V	N	A	S	I	S	N	G	F	G	-	A	A	A	T	D	Y	T	Y	N	K	A	T	N	D	F	T	F	N	A	S	I	A	A	A	A	-	V	G	D
Translation of O145 RM12581	L	A	D	G	A	V	V	N	A	S	I	S	N	G	F	G	-	A	A	A	T	D	Y	T	Y	N	K	A	T	N	D	F	T	F	N	A	S	I	A	A	A	A	-	V	G	D
Translation of O28 CB9651	L	A	D	G	A	V	V	N	A	S	I	S	N	G	F	G	-	A	A	A	T	D	Y	T	Y	N	K	A	T	N	D	F	T	F	N	A	S	I	A	A	A	A	-	V	G	D
Translation of O152 SE11	L	A	N	N	A	K	V	N	A	T	I	A	N	G	F	G	S	P	T	A	T	D	Y	T	Y	N	S	A	T	G	D	F	T	Y	S	A	T	I	A	A	G	T	N	S	G	D
Translation of O132 N87	L	A	N	N	A	K	V	N	A	T	I	A	N	G	F	G	S	P	T	A	T	D	Y	T	Y	N	S	A	T	G	D	F	T	Y	S	A	T	I	A	A	G	T	N	S	G	D
Translation of O26:H46 5306-56	L	A	D	G	A	V	V	D	A	S	I	S	N	G	F	G	A	A	A	A	T	D	Y	T	Y	N	K	A	T	N	D	F	T	F	N	A	S	I	A	A	G	A	A	A	G	D
	L	A	d	g	A	v	V	n	A	s	I	s	N	G	F	G	s	a	a	A	T	D	Y	T	Y	N	k	A	T	n	D	F	T	f	n	A	s	I	A	A	X	a	n	X	G	D

Alignment Name: Translation of H28 and H46 strains.aaa_bsml
Length: 585

280 290 300 310 320

Translation of O145 CB12513	SNSAALQSFLTPKAGDTANLSVKIGTTSVDVVLASDGKITAKDGSE
Translation of O145 RM12581	SNSAALQSFLTPKAGDTANLSVKIGTTSVDVVLASDGKITAKDGSE
Translation of O28 CB9651	SNSAALQSFLTPKAGDTANLSVKIGTTSVDVVLASDGKITAKDGSE
Translation of O152 SE11	SNSAQLQSFLTPKAGDTANLNVKIGSTSIDVVLASDGKITAKDGSE
Translation of O132 N87	SNSAQLQSFLTPKAGDTANLNVKIGSTSIDVVLASDGKITAKDGSE
Translation of O26:H46 5306-56	SNSAALQSFLTPKAGDTANLSVKIGTTSVNVVLASDGKITAKDGSA
	SNSAaLQSFLTPKAGDTANLsVKIGtTSvdVVLASDGKITAKDGSe

330 340 350 360

Translation of O145 CB12513	LFIDVDGNLTQNNAGTVKAATLDALTKNWHTTGTPDAVSTVITTED
Translation of O145 RM12581	LFIDVDGNLTQNNAGTVKAATLDALTKNWHTTGTPDAVSTVITTED
Translation of O28 CB9651	LFIDVDGNLTQNNAGTVKAATLDALTKNWHTTGTPDAVSTVITTED
Translation of O152 SE11	LFIDVDGNLTQNNAGTVKAATLDALTKNWHTTGTPGAVSTVITTED
Translation of O132 N87	LFIDVDGNLTQNNAGTVKAATLDALTKNWHTTGTPSAVSTVITTED
Translation of O26:H46 5306-56	LYIDSTGNLTQNSAGTVTAATLDGLTKNHDATG---AVGVDITTAD
	LfIDvdGNLTQNnAGTVkAATLDaLTKNwhtTGTPdAVstvITTeD

370 380 390 400 410

Translation of O145 CB12513	ETTFTLAGGTDATT---SGTITVANARMSAESLQSATKSTGFTVDV
Translation of O145 RM12581	ETTFTLAGGTDATT---SGTITVANARMSAESLQSATKSTGFTVDV
Translation of O28 CB9651	ETTFTLAGGTDATT---SGTITVANARMSAESLQSATKSTGFTVDV
Translation of O152 SE11	ETTFTLAGGTDATT---SGAITVANARMSAESLQSATKSTGFTVDV
Translation of O132 N87	ETTFTLAGGTDATT---SGAITVANARMSAESLQSATKSTGFTVDV
Translation of O26:H46 5306-56	GATISLAGSANAATGTQSGAITLKNVRISADALQSAAKGTVINVDN
	etTftLAGgtdAtTGTQSGXITvaNaRmSAesLQSAtKsTgftVDv

Alignment Name: Translation of H28 and H46 strains.aaa_bsml
Length: 585

						420										430										440										450										460
Translation of O145 CB12513	G	T	T	G	T	S	A	G	D	I	K	V	D	S	K	G	I	V	Q	Q	H	T	G	T	G	F	E	D	A	Y	T	K	A	D	G	S	L	T	T	D	N	T	T	N	L	F
Translation of O145 RM12581	G	T	T	G	T	S	A	G	D	I	K	V	D	S	K	G	I	V	Q	Q	H	T	G	T	G	F	E	D	A	Y	T	K	A	D	G	S	L	T	T	D	N	T	T	N	L	F
Translation of O28 CB9651	G	T	T	G	T	S	A	G	D	I	K	V	D	S	K	G	I	V	Q	Q	H	T	G	T	G	F	E	D	A	Y	T	K	A	D	G	S	L	T	T	D	N	T	T	N	L	F
Translation of O152 SE11	G	A	T	G	N	S	A	G	D	I	K	V	D	S	K	G	I	V	Q	Q	Y	T	G	T	V	F	E	D	A	Y	T	K	A	D	G	S	L	T	T	D	N	T	T	N	L	F
Translation of O132 N87	G	A	T	G	T	S	A	G	D	I	K	V	D	S	K	G	I	V	Q	Q	H	T	G	T	G	F	E	D	A	Y	T	K	A	D	G	S	L	T	T	D	N	T	T	N	L	F
Translation of O26:H46 5306-56	G	-	-	-	-	-	A	D	D	I	S	V	S	K	T	G	V	V	T	T	G	G	-	-	-	-	A	P	T	Y	T	D	A	D	G	K	L	T	T	T	N	T	V	D	Y	F
	G	t	T	G	t	S	A	g	D	I	k	V	d	s	k	G	i	V	q	q	h	t	G	T	g	F	e	d	a	Y	T	k	A	D	G	s	L	T	T	d	N	T	t	n	l	F

										470										480										490										500						
Translation of O145 CB12513	L	Q	K	D	G	T	V	T	N	G	S	G	K	A	V	Y	V	S	A	D	G	N	F	T	T	D	A	E	T	K	A	A	T	T	A	D	P	L	K	A	L	D	E	A	I	S
Translation of O145 RM12581	L	Q	K	D	G	T	V	T	N	G	S	G	K	A	V	Y	V	S	A	D	G	N	F	T	T	D	A	E	T	K	A	A	T	T	A	D	P	L	K	A	L	D	E	A	I	S
Translation of O28 CB9651	L	Q	K	D	G	T	V	T	N	G	S	G	K	A	V	Y	V	S	A	D	G	N	F	T	T	D	A	E	T	K	A	A	T	T	A	D	P	L	K	A	L	D	E	A	I	S
Translation of O152 SE11	L	Q	K	D	G	T	V	T	N	G	S	G	K	A	V	Y	V	S	A	D	G	N	F	T	T	D	A	E	T	K	A	A	T	T	A	D	P	L	K	A	L	D	E	A	I	S
Translation of O132 N87	L	Q	K	D	G	T	V	T	N	G	S	G	K	A	V	Y	V	S	A	D	G	N	F	T	T	D	A	E	T	K	A	A	T	T	A	D	P	L	K	A	L	D	E	A	I	S
Translation of O26:H46 5306-56	L	Q	T	D	G	S	V	T	N	G	S	G	K	G	V	Y	T	D	A	A	G	K	F	T	T	D	A	A	T	K	A	A	T	T	T	D	P	L	K	A	L	D	D	A	I	S
	L	Q	k	D	G	t	V	T	N	G	S	G	K	a	V	Y	v	s	A	d	G	n	F	T	T	D	A	e	T	K	A	A	T	T	a	D	P	L	K	A	L	D	e	A	I	S

				510										520										530										540										550		
Translation of O145 CB12513	S	I	D	K	F	R	S	S	L	G	A	V	Q	N	R	L	D	S	A	V	T	N	L	N	N	T	T	T	N	L	S	E	A	Q	S	R	I	Q	D	A	D	Y	A	T	E	V
Translation of O145 RM12581	S	I	D	K	F	R	S	S	L	G	A	V	Q	N	R	L	D	S	A	V	T	N	L	N	N	T	T	T	N	L	S	E	A	Q	S	R	I	Q	D	A	D	Y	A	T	E	V
Translation of O28 CB9651	S	I	D	K	F	R	S	S	L	G	A	V	Q	N	R	L	D	S	A	V	T	N	L	N	N	T	T	T	N	L	S	E	A	Q	S	R	I	Q	D	A	D	Y	A	T	E	V
Translation of O152 SE11	S	I	D	K	F	R	S	S	L	G	A	V	Q	N	R	L	D	S	A	V	T	N	L	N	N	T	T	T	N	L	S	E	A	Q	S	R	I	Q	D	A	D	Y	A	T	E	V
Translation of O132 N87	S	I	D	K	F	R	S	S	L	G	A	V	Q	N	R	L	D	S	A	V	T	N	L	N	N	T	T	T	N	L	S	E	A	Q	S	R	I	Q	D	A	D	Y	A	T	E	V
Translation of O26:H46 5306-56	Q	I	D	K	F	R	S	S	L	G	A	I	Q	N	R	L	D	S	A	V	T	N	L	N	N	T	T	T	N	L	S	E	A	Q	S	R	I	Q	D	A	D	Y	A	T	E	V
	s	I	D	K	F	R	S	S	L	G	A	v	Q	N	R	L	D	S	A	V	T	N	L	N	N	T	T	T	N	L	S	E	A	Q	S	R	I	Q	D	A	D	Y	A	T	E	V

Alignment Name: Translation of H28 and H46 strains.aaa_bsml
Length: 585

								560										570										580					
Translation of O145 CB12513	S	N	M	S	K	A	Q	I	I	Q	Q	A	G	N	S	V	L	A	K	A	N	Q	V	P	Q	Q	V	L	S	L	L	Q	G
Translation of O145 RM12581	S	N	M	S	K	A	Q	I	I	Q	Q	A	G	N	S	V	L	A	K	A	N	Q	V	P	Q	Q	V	L	S	L	L	Q	G
Translation of O28 CB9651	S	N	M	S	K	A	Q	I	I	Q	Q	A	G	N	S	V	L	A	K	A	N	Q	V	P	Q	Q	V	L	S	L	L	Q	G
Translation of O152 SE11	S	N	M	S	K	A	Q	I	I	Q	Q	A	G	N	S	V	L	A	K	A	N	Q	V	P	Q	Q	V	L	S	L	L	Q	G
Translation of O132 N87	S	N	M	S	K	A	Q	I	I	Q	Q	A	G	N	S	V	L	A	K	A	N	Q	V	P	Q	Q	V	L	S	L	L	Q	G
Translation of O26:H46 5306-56	S	N	M	S	K	A	Q	I	I	Q	Q	A	G	N	S	V	L	A	K	A	N	Q	V	P	Q	Q	V	L	S	L	L	Q	G
	S	N	M	S	K	A	Q	I	I	Q	Q	A	G	N	S	V	L	A	K	A	N	Q	V	P	Q	Q	V	L	S	L	L	Q	G
